# Persistence and Exposure Assessment of Insecticide Indoxacarb Residues in Vegetables

**DOI:** 10.3389/fnut.2022.863519

**Published:** 2022-05-09

**Authors:** Sandip Patra, Anupam Das, Rajiv Rakshit, Suborna Roy Choudhury, Shyamashree Roy, Tilak Mondal, Arunava Samanta, Pritam Ganguly, Amnah Mohammed Alsuhaibani, Ahmed Gaber, Marian Brestic, Milan Skalicky, Akbar Hossain

**Affiliations:** ^1^Division of Crop Science, ICAR Research Complex for NEH Region, Shillong, India; ^2^Department of Soil Science & Agricultural Chemistry, Bihar Agricultural University, Bhagalpur, India; ^3^Department of Agronomy, Bihar Agricultural University, Bhagalpur, India; ^4^Department of Agronomy, RRS (OAZ), Uttar Banga Krishi Viswavidyalaya, Majhian, India; ^5^Department of Crop Production, ICAR – Vivekananda Parvatiya Krishi Anusandhan Sansthan, Almora, India; ^6^Department of Agricultural Entomology, Bidhan Chandra Krishi Viswavidyalaya, Mohanpur, India; ^7^Department of Physical Sport Science, College of Education, Princess Nourah bint Abdulrahman University, Riyadh, Saudi Arabia; ^8^Department of Biology, College of Science, Taif University, Taif, Saudi Arabia; ^9^Department of Plant Physiology, Slovak University of Agriculture, Nitra, Slovakia; ^10^Department of Botany and Plant Physiology, Faculty of Agrobiology, Food, and Natural Resources, Czech University of Life Sciences Prague, Prague, Czechia; ^11^Department of Agronomy, Bangladesh Wheat and Maize Research Institute, Dinajpur, Bangladesh

**Keywords:** indoxacarb, vegetables, soil, persistence, Risk Assessment

## Abstract

Indoxacarb, a promising new generation insecticide, is gaining popularity among vegetable growers in West Bengal, India, for controlling a large number of insects. However, it may simultaneously also increase the risk of contamination in the edible portions of the vegetables. This study was planned to analyze the persistence behavior of indoxacarb in cabbages, tomatoes, and soil. Moreover, indoxacarb residue contents were estimated to assess both the dietary and soil ecological risks associated with the application of the same. The experimental location was important because West Bengal is the leading vegetables producing state in India. Indoxacarb was found to dissipate quickly with a half-life ranging between 1.55 and 2.76 days, irrespective of the vegetable, dose, and season, and the safe waiting period was very less. The findings indicate that both vegetables can be safely consumed 1 day after the final spray. However, the risk to soil algae is predicted to be unacceptably high, which needs to be studied extensively.

## Introduction

Vegetables are one of the most important ingredients in our daily diet because they serve as good sources of vitamins, minerals, amino acids, and fiber. It is recommended that we need to consume an average of 120–140 g of vegetables daily for maintaining good health (1). Two important vegetables, namely cabbages and tomatoes, were chosen for this study for two main reasons: (i) the intensive application of insecticide as a result of heavy insect-pest pressure on these crops, and (ii) both vegetables are consumed raw in salads as well as cooked.

Cabbage (*Brassica oleracea* L. var. *capitata*) is an important cruciferous vegetable grown throughout the world. Globally, India ranks second in vegetable production. The crop covers an area of around 4.07 million hectares, with a total production of around 89.7 million tons annually. West Bengal is the highest cabbage-producing state in India, contributing around 28.2% to the total production of the country (2). Cabbage is a good source of β-carotene, carbohydrates, and fibers, and it is also rich in several minerals and vitamins like A, B1, B2, and C (3). This vegetable contains an indole group of substances which can prevent stomach and colon cancer in humans (4). In India, about 20–70% yield losses occur with cabbage as a result of lepidopteran insect infestations (5). Among the lepidopteran insect pests such as *Plutella xylostella* Linnaeus, *Pieris brassicae* Linnaeus, *Thysanoplusia orichalcea* Fabricius, and *Spodoptera litura* are the most important.

Tomatoes (*Lycopersicon esculentum* Mill.) are another important vegetable cultivated in India. The crop covers almost double the area of cabbage crops, at around 8.09 million hectares, with a total production of approximately 196.9 million tons annually (2). Tomatoes contain high amounts of nutrient minerals, carbohydrates, proteins, and vitamins, mainly vitamin C. They are also a very good source of lycopene, which can play an important role in reducing cardiovascular diseases and cancer (6). Tomatoes are very susceptible to infestation by insects such as *Helicoverpa armigera, Bemisia tabaci, Spodoptera litura*, and *Liriomyza trifolii*, which cause huge crop damage in the field (7). It was reported that 25–70% yield losses with tomatoes happen as a result of infestation by fruit borers alone in India (5).

**Figure 1:**
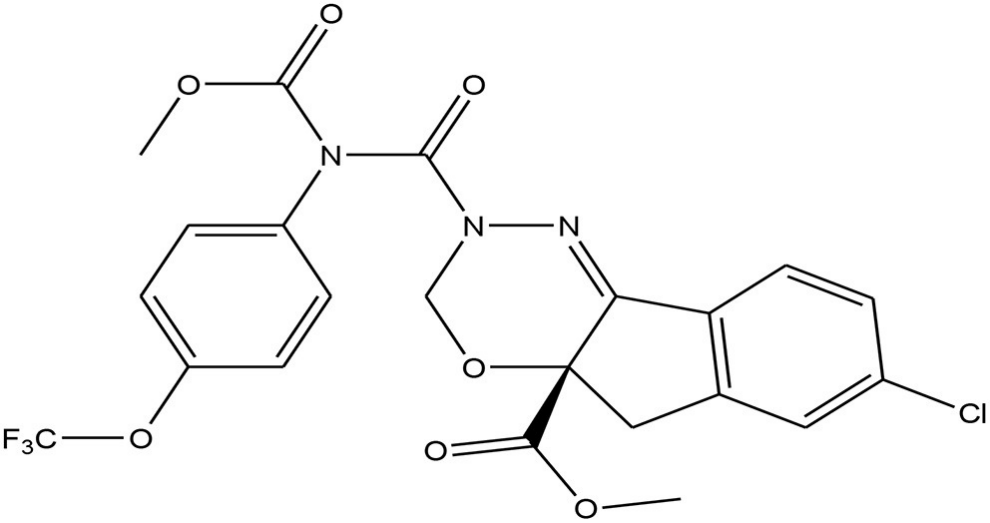
Chemical structure of Indoxacarb.

In modern agricultural farming practices, pesticides are essential for protecting crops from diseases and insect-pest infestations. Among the new generation insecticides, indoxacarb ((*S*)-methyl 7-chloro-2,5-dihydro-2-{[(methoxycarbonyl)[4-(trifluoromethoxy)phenyl]amino]carbonyl}indeno[1,2-e] (1, 3, 4) oxadiazine-4a(3*H*)-carboxylate [C_22_H_17_ClF_3_N_3_O_7_)] (Figure 1) is one that is highly effective against lepidopteran insect pests (8, 9). It is in the oxadiazine group of insecticides and has broad-spectrum activity.

Indoxacarb has been used to control lepidopteran insect pests on edible fruits, vegetables, and fiber crops like cotton (10, 11) especially fruit borers and leaf folders on chillies (12), fruit borers on tomatoes (13), diamondback moths (14, 15), and cabbage loopers (9). It blocks the sodium channel in insect neurons (16) and is categorized as a reduced risk insecticide by the United States Environmental Protection Agency (USEPA) (17). It has slight side effects on non-target insects (18, 19) and is comparatively safe for most predators and immature wasp parasites (20, 21). However, the wet residues of indoxacarb can be toxic to honey bees and adult wasp parasites.

The dissipation pattern of indoxacarb has been studied in cabbages (22), cucumbers, tomatoes, apples, pears and soil (23), cauliflowers (24), brinjal (also known as aubergine or eggplant) (25), and okra (26) in different regions. As indoxacarb is extensively used in different crops, the safety parameters for the consumption of raw vegetables treated with this insecticide need to be examined. This study aims to generate meaningful information about the persistence of indoxacarb residues in both vegetables and soil. We assess the safety risk for human beings pertaining to the consumption of these vegetables and for the soil habitats as well. A simple, robust, and precise analytical method is developed and validated based on standard protocols and may be followed in the future for further investigations. The experimental location is situated in West Bengal (Eastern India), which is the leading state for vegetable production (27) and contributes 15% to the total national vegetable production (2). However, information regarding the residual behavior of indoxacarb under agro-climatic conditions of West Bengal-India is not available yet. Therefore, this study is focused on the persistence and dissipation behavior of indoxacarb residues in/on cabbages and tomatoes, with a dietary safety assessment.

## Materials and Methods

### Chemicals and Reagents

A reference standard of analytical-grade indoxacarb (99.8%) was supplied by Gharda Chemicals Limited, India. Indoxacarb 14.5% SC formulation was obtained from Devidayal Agro Chemicals, India. All solvents and reagents were of analytical grade. All solvents utilized in the experiment were glass distilled before use. The reagents, namely anhydrous sodium sulfate, neutral alumina, and sodium chloride, were activated prior to use and kept in desiccators.

### Preparation of Standard Solutions

A stock solution (1,000 μg ml^−1^) of the indoxacarb analytical standard was made by accurately weighing 10 mg (±0.01 mg, purity 99.8%) into a certified ‘A' class volumetric flask and dissolving it in 10 ml of ethyl acetate; the solution was stored in a refrigerator at 4°C. A working standard mixture of 100 μg ml^−1^ was made by diluting the stock solution ten times. Calibration standards ranging between 0.01 and 1.0 μg ml^−1^were made by serial dilutions with ethyl acetate.

### Field Experiments

Field experiments were carried out at the Research Farm of Bidhan Chandra Krishi Viswavidyalaya, Kalyani, Nadia, West Bengal, India, to study the dissipation behavior and estimate the indoxacarb residues in cabbages, tomatoes, and soil. The experimental site was located at an altitude of 11 m, the latitude of 22.99°N and longitude of 88.43°E. Cabbage (cv. Rareball) and tomato (cv. Nidhi) crops were grown for two consecutive seasons by following good agricultural practices (GAP). Indoxacarb was applied at the recommended dose (T_1_: 75 g a.i. ha^−1^) and double the recommended dose (T_2_: 150 g a.i. ha^−1^) in both crops. Indoxacarb was applied twice with an interval of 15 d, starting from the head-formation stage for cabbages and the fruiting stage for tomatoes, by using a knapsack sprayer. Untreated control plots (T_3_: water spray only) were maintained for both crops. Each experimental plot was 12 m^2^ (4 m × 3 m) in size, with three replications of each. The total rainfall amounts received during tomato cultivation were 113.00 mm in season I and none in season II, whereas the corresponding values during cabbage cultivation were 9.00 mm and 28.50 mm, respectively.

### Sampling

About 4–5 marketable-size tomato fruits and 2–3 medium-size cabbage heads were sampled on days 0 (2 h), 1, 3, 5, 7, 10, and 14 after the final application of indoxacarb. The samples were taken from each plot separately in a random fashion. Soil samples were taken separately from each plot by using a tube auger at a depth of approximately 10–15 cm on the same days as plant sampling and at harvest. The soil samples from the different sites were pooled and sieved through a 2 mm mesh sieve, air-dried, and processed for further residual analysis. The texture and characteristics of the soil samples were sand 71.0%, silt 16.4%, clay 12.6%, organic carbon 0.65%, electrical conductivity 0.25 ds m^−1^, and pH 6.92. Samples were processed immediately after collection.

### Extraction and Clean-Up of Cabbage, Tomato, and Soil Samples

Cabbage and tomato fruits were chopped into small pieces, and 50 g representative samples were drawn by the quartering method. Each sample was then kept in a 250 ml conical flask for 2 h with 100 ml of a mixture of distilled acetone and water (8:2, v/v). The sample was then homogenized in a homogenizer at 10,000 rpm for 5 min and transferred to a wide-mouthed conical flask. The sample was filtered through a Whatman No. 1 filter paper by using a Buchner funnel. The solid portion of the sample was poured back into the jar, and the extraction process was repeated twice (50 ml + 50 ml) with distilled acetone and water (8:2, v/v) each time. The combined filtrates were subjected to rotary vacuum evaporation at 40°C to completely remove the acetone. The concentrated extract was then transferred to a 500 ml separatory funnel. The evaporating flask was rinsed twice with dichloromethane (25 ml + 25 ml), and the organic solvent was added to the separatory funnel. After that, 100 ml of 10% aqueous NaCl was added to the extract, and the mixture was shaken vigorously for 20–30 s. After the layers separated, the organic phase was collected in a flask, and the process of partition was repeated twice (50 ml + 50 ml) with dichloromethane. The organic phases were then combined, the volume was reduced to dryness by using a rotary vacuum evaporator at 40°C and the residue was reconstituted in 5 ml of hexane.

The extract was cleaned by adsorption column chromatography with neutral alumina as the adsorbent. First, 10 g of neutral alumina was placed between two anhydrous Na_2_SO_4_ (2 g) layers by using 50 ml of distilled hexane as the packing solvent. The sample was loaded into the column, and the column was washed with a mixture of 10% acetone and hexane (50 ml). All of the eluents used so far were discarded, and the column was finally eluted with 100% acetone (100 ml). The final eluent was immediately concentrated to dryness by using a rotary vacuum evaporator at 40°C. The residue was reconstituted in 10 ml of ethyl acetate for gas chromatography (GC) analysis with electron-capture detection (ECD).

Soil samples from cabbage and tomato fields were brought to the laboratory for immediate processing, extraction, and clean-up. A total of 50 g of soil was placed in a 250 ml conical flask. A mixture of acetone and water (100 ml, 8:2, v/v) was then added, and the mixture was left for 2 h. The extraction and clean-up of soil samples were performed by following the same method as that used for plant samples (tomato and cabbage).

### GC–ECD Estimation

Residues of indoxacarb in cabbage, tomato, and field soil were analyzed by using an Agilent 6890N gas chromatograph equipped with an electron-capture detector and Chemstation software. The instrument was equipped with an automatic sampler (G-2614A), and a wide-bore HP-5 column (internal diameter: 0.32 mm; length: 32 m) was installed in it. During analysis, the flow rate of 2 ml min^−1^, the film thickness of 0.25 μm, and a split ratio of 10:1 were maintained. Highly pure nitrogen gas was used as the carrier gas. The oven temperature was set to 180°C, held, and then increased up to 280°C at 10°C min^−1^ and held for 3 min. The injection temperature of 280°C, detector temperature of 300°C, and post-run temperature of 310°C for 5 min were maintained, and the injection volume was 2 μl.

### Method Validation

The present analytical method was validated based on SANTE guidelines (28). Validations of different parameters were performed in cabbage, tomato, and soil matrices. Various validation parameters were included in the study, such as linearity, trueness (recovery), precision, sensitivity, specificity, and matrix effect. Six levels of indoxacarb matrix-matched standards, ranging between 0.01 and 0.30 μg ml^−1^, were injected into the instrument for obtaining peak areas. A calibration curve was constructed on this basis to judge the linearity of the method. Specificity was determined as the percentage of the average peak area of the blank sample relative to that of a blank sample with added indoxacarb standard, with six replications of each. Blank samples fortified with 0.03, 0.15, and 0.30 μg ml^−1^ concentrations of indoxacarb and replicated three times were processed as per the method to evaluate the recovery (trueness). The recovery efficiency (RE) was calculated by dividing the detected average residues by the respective spiked level and multiplying by 100. The relative standard deviation (RSD) values of each spiked replicate were considered for estimating the repeatability. The Horwitz ratio (HorRat) was calculated based on the following formula for evaluating the intra-laboratory precision (reproducibility) of the method (29, 30).


$$\begin{array}{l} \text{HorRat}=\text{RSD/PRSD} \end{array}$$

In which the predicted RSD (PRSD) equals 2C^−0.15^, and C represents the concentration expressed as a mass fraction (10 ng g^−1^ = 10 × 10^−9^).

Both of these values were a measure of the precision of the analytical method. The limit of detection (LOD) of the analyte was calculated by considering a signal-to-noise ratio of 3, whereas the corresponding value for the limit of quantification (LOQ) values calculated was 10. Both of these LOD and LOQ values were a measure of the sensitivity of the analytical method. The matrix effect (ME) of the method was determined by using the following formula to avoid erroneous reports (false positive/false negative).

ME (%) = [peak area of post-extraction spiking/peak area of the solvent standard] × 100 (31).

Another important parameter, known as process efficiency (PE), was also evaluated by using the following formula (31).


$$\begin{array}{l} \text{PE (\%)}\;=\;\left[ME\;\times \;RE\right]/100 \end{array}$$

### Dissipation of Indoxacarb

The dissipation of indoxacarb is subject to first-order kinetics (32), and the regression equation can be expressed by using the following formula.


$$\begin{array}{l} C_{\text{t}}=C_{0}e^{-kt} \end{array}$$

In which C_t_ represents the residue content (μg g^−1^) at time t (d) after insecticide application, k denotes the dissipation rate constant and C_0_ represents the initial deposit content (μg g^−1^).

The residual half-life (RL_50_) in days was calculated based on the following equation.


$$\begin{array}{l} {\text{RL}}_{50}\;=\;ln\;2/K \end{array}$$

In which *K* is the slope of the regression line.

### Pre-harvest Interval (PHI)

The PHI can be defined as the minimum time interval (in days) required between the final application of pesticide and the harvest to allow the pesticide residues to fall below the maximum residue limit (MRL). It was calculated by using the following equation (27).


$$\begin{array}{l} \text{PHI}\;=\;\left[ln\;A-\;ln\;\left(MRL\right)\right]/K \end{array}$$

In which *A* is the amount of initial deposit (μg g^−1^) and *K* is the slope of the regression line. In India, the MRL values for indoxacarb are fixed as 0.50 and 3.0 μg g^−1^ in tomato and cabbage crops, respectively (33).

### Safety Risk Assessment

#### Dietary Risk Assessment

The estimated daily intake (EDI) of indoxacarb through consumption of tomatoes and cabbages was measured by multiplying the residue content found in the vegetables by the recommended dietary consumption per day (34). The long-term dietary risk quotient (RQ_d_) was determined by the following equation:

RQ_d_= EDI/(ADI × average body weight),

In which ADI represents the acceptable daily intake.

The appropriate ADI value for indoxacarb is 0.006 mg kg^−1^ of body weight d^−1^ (35). The recommended daily vegetable consumption and average body weight of an Indian adult were considered to be 300 g (36) and 55 kg (37), respectively. If the RQ_d_ value is found to be greater than 1, there may be a considerable risk of indoxacarb toxicity. The EDI value was considered to be half of the LOQ in cases when indoxacarb was not detected in the sample (38). For assessment of acute toxicity, the residue content of indoxacarb was compared with the corresponding acute reference dose value, that is, 0.125 mg kg^−1^ body weight (35) multiplied by the average body weight.

#### Risk Assessment Pertaining to Soil Flora and Fauna

The risk quotient (RQ_s_) values of indoxacarb pertaining to representative soil flora and fauna were determined by the process described in the Technical Guidance Document on Risk Assessment (39). In this study, acute 72 h EC_50_ (0.079 mg L^−1^) and acute 14 d LC_50_ (625 mg kg^−1^) values were considered for algae and earthworms, respectively. The predicted no-effect concentration (PNEC) values were derived by dividing the respective toxicity values by 1,000, that is, the assessment factor for this case. The RQ_s_ values were then calculated by using the following formula: RQ_s_ = EC/PNEC (40), in which EC stands for effective concentration of pesticide, that is, indoxacarb in soil for this case. The risk may be predicted to be low if the RQ_s_ value is found to be <1. If the value comes between 0.1 and 1.0, the risk may be predicted to be moderate. If the RQ_s_ value is found to be > 1, there may be an unacceptably high risk because of the presence of indoxacarb residues in the soil. The EC value was considered on behalf of the LOQ if indoxacarb is not detected in the sample (38).

## Results and Discussion

### Method Validation

**Figure 2:**
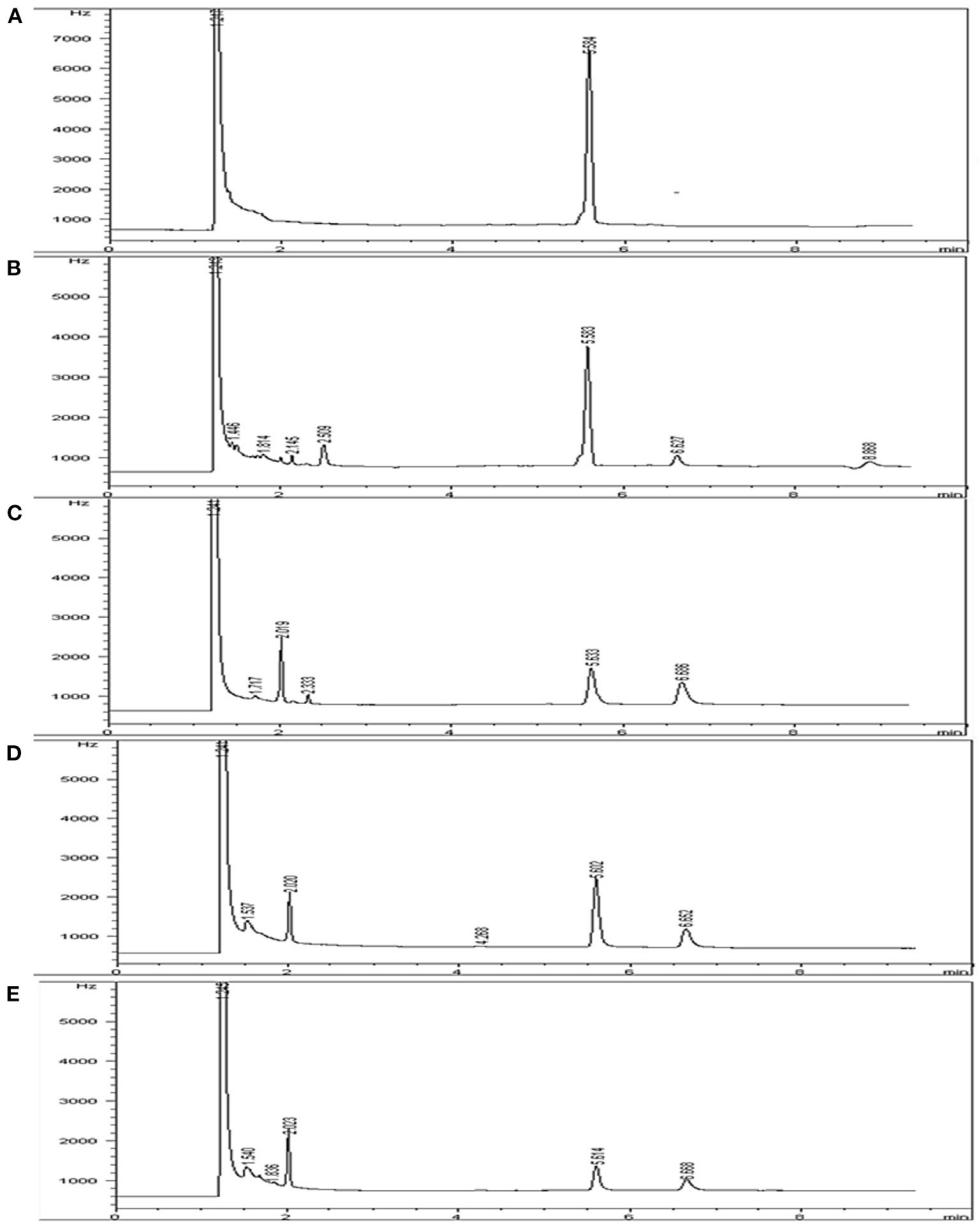
Chromatogram of **(A)** indoxacarb standard solution, **(B)** cabbage sample, **(C)** cabbage field soil sample, **(D)** tomato sample, and **(E)** tomato field soil sample.

Indoxacarb residue GC peaks were detected at 5.60 ± 0.10 min in different substrates under set conditions with acceptable specificity. The indoxacarb residues were identified by comparing the retention time of the sample peak with that of the analytical standard. Chromatograms of the indoxacarb standard solution, cabbage sample, tomato sample, and field soil samples are shown in Figure 2.

**Figure 3:**
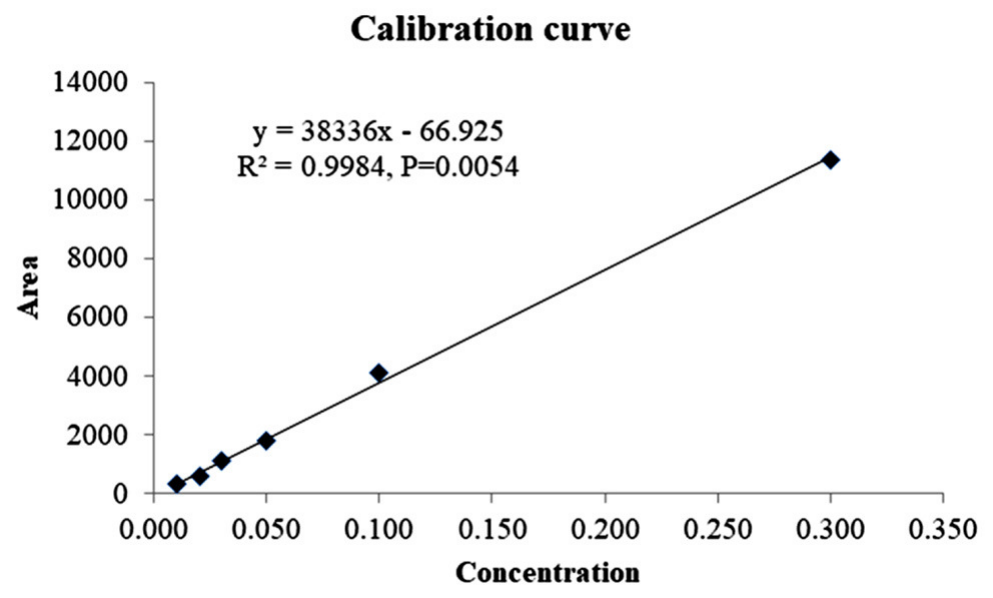
Calibration curve of indoxacarb.

The analytical method was found to be linear in the range between 0.01 and 0.30 μg ml^−1^ as the correlation coefficient was recorded as 0.9984 (Figure 3). The LOD and LOQ values were found to be 0.01 and 0.03 μg g^−1^, respectively, in each substrate. Therefore, the method was sensitive enough because the LOQ value is well below the MRL value of indoxacarb for both vegetables. Different method validation parameters were evaluated and are presented in Table 1. Recovery of the analyte from different substrates was found to be satisfactory because the RE and RSD values were in the acceptable ranges, i.e., 83.33–90.67% and ≤ 13.09%, respectively. The HorRat values of the different substrates were in the acceptable range of 0.5–2.0 (30). The matrix effects were also found to be acceptable because the values were well below 120% (28). The overall process efficiency was very encouraging, with values > 90%. Thus, the present method fulfills the criteria laid down in the SANTE guidelines and is fit for the analysis.

**Table 1:** Results of method validation of indoxacarb.

**Substrate**	**Spiked level (μg g^**−1**^)**	**Indoxacarb**							
		**Mean Residue (μg g^**−1**^)**	**SD**	**RSD (%)**	**PRSD**	**HorRat**	**RE (%)**	**ME (%)**	**PE (%)**
Tomato	0.03	0.03	0.00	11.89	9.75	1.22	86.67	107.44	93.11
	0.15	0.13	0.01	9.80	7.64	1.28	87.44	108.85	95.18
	0.30	0.27	0.03	11.78	6.85	1.72	91.17	104.78	95.52
Cabbage	0.03	0.03	0.00	13.09	9.80	1.34	83.33	110.05	91.71
	0.15	0.13	0.01	9.12	7.63	1.20	88.78	105.97	94.08
	0.30	0.27	0.03	9.57	6.86	1.40	89.89	105.78	95.08
Soil	0.03	0.03	0.00	8.27	9.75	0.85	86.67	105.39	91.34
	0.15	0.13	0.01	9.89	7.66	1.29	86.00	108.13	92.99
	0.30	0.27	0.03	10.38	6.85	1.51	90.67	106.81	96.84

### Dissipation Behavior of Indoxacarb in/on Tomatoes, Cabbages, and Soil

The results pertaining to the dissipation pattern of indoxacarb residues in/on the two vegetables and soil are presented in Table 2.

**Table 2:** The residue of indoxacarb in different substrates.

**Day**	**Dose**	**Residue of indoxacarb (μg g** ^**−1**^ **)**							
		**Tomato**		**Soil**		**Cabbage**		**Soil**	
		**Season I**	**Season II**	**Season I**	**Season II**	**Season I**	**Season II**	**Season I**	**Season II**
0	T_1_	0.92 ± 0.04	0.93 ± 0.14	0.84 ± 0.04	0.86 ± 0.05	1.49 ± 0.05	1.58 ± 0.06	1.30 ± 0.06	1.35 ± 0.07
	T_2_	1.60 ± 0.04	1.65 ± 0.12	1.53 ± 0.07	1.61 ± 0.08	2.62 ± 0.03	2.58 ± 0.17	2.32 ± 0.15	2.43 ± 0.11
1	T_1_	0.51 ± 0.03	0.75 ± 0.11	0.47 ± 0.06	0.43 ± 0.04	1.23 ± 0.05	1.15 ± 0.07	0.72 ± 0.04	0.67 ± 0.05
	T_2_	0.80 ± 0.05	1.13 ± 0.11	0.92 ± 0.06	1.03 ± 0.05	2.06 ± 0.04	1.98 ± 0.02	1.14 ± 0.08	1.05 ± 0.11
3	T_1_	0.20 ± 0.03	0.38 ± 0.10	0.25 ± 0.06	0.32 ± 0.02	0.69 ± 0.04	0.61 ± 0.04	0.38 ± 0.03	0.32 ± 0.07
	T_2_	0.30 ± 0.04	0.66 ± 0.11	0.58 ± 0.09	0.69 ± 0.02	1.07 ± 0.05	1.08 ± 0.12	0.64 ± 0.05	0.56 ± 0.04
7	T_1_	0.04 ± 0.01	0.17 ± 0.07	0.13 ± 0.05	0.20 ± 0.02	0.22 ± 0.04	0.18 ± 0.03	0.14 ± 0.03	0.08 ± 0.01
	T_2_	0.08 ± 0.01	0.31 ± 0.10	0.23 ± 0.02	0.44 ± 0.04	0.39 ± 0.02	0.29 ± 0.04	0.28 ± 0.03	0.17 ± 0.02
10	T_1_	-	0.05 ± 0.02	0.05 ± 0.03	0.09 ± 0.01	0.06 ± 0.01	0.04 ± 0.01	0.05 ± 0.01	0.04 ± 0.00
	T_2_	-	0.17 ± 0.10	0.10 ± 0.02	0.17 ± 0.02	0.11 ± 0.01	0.22 ± 0.12	0.09 ± 0.02	0.07 ± 0.02
14	T_1_	-	-	-	-	-	-	-	-
	T_2_	-	0.04 ± 0.01	0.05 ± 0.01	0.09 ± 0.00	0.05 ± 0.00	0.03 ± 0.00	-	-
Half-life (d)	T_1_	1.55	2.46	2.75	3.49	2.19	1.90	2.26	1.99
	T_2_	1.67	2.76	2.83	3.55	2,38	2.34	2.36	2.11
PHI (d)	T_1_	1.37	2.21	-	-	-	-	-	-
	T_2_	2.81	4.76	-	-	-	-	-	-
Regression Equation	T_1_	y = 6.736–0.4467x	y = 6.8683–0.2823x	y = 6.5084–0.2516x	y = 6.5014–0.1986x	y = 7.4193–0.317x	y = 7.4525–0.3648x	y = 6.9977–0.3073x	y = 6.9648–0.3486x
	T_2_	y = 7.1792–0.4157x	y = 7.3679–0.2513x	y = 7.166–0.2451x	y = 7.2456–0.195x	y = 7.8777–0.2909x	y = 7.8935–0.296x	y = 7.5146–0.2935x	y = 7.4843–0.3292x
Correlation coefficient (R^2^)	T_1_	0.9963	0.9872	0.9338	0.9424	0.9891	0.9842	0.9893	0.9854
	T_2_	0.9773	0.9833	0.9941	0.9812	0.9912	0.9796	0.9764	0.9773

*ND, Not Detected at LOQ of 0.03 μg g^−1^*.

The initial deposit of indoxacarb in cabbages was found to be slightly higher than that in tomatoes, irrespective of the dose and season, which may be the result of a larger exposure area (cabbage head). Residues dissipated quickly in both vegetables and were not detectable within 14 d of the final spray of insecticide applied at the recommended dose. The RL_50_ values of indoxacarb were in the range of 1.55–2.76 d, irrespective of the vegetable, dose, and season, which indicates that the compound is less persistent in the crop. In another experiment with cabbages, the compound showed a half-life of 2.88 d when applied at 52.2 g a.i. ha^−1^ and of 1.92 d at 104.4 g a.i. ha^−1^ (22). A similar result was reported with half-life values of 1.6–2.3 d in eggplant crops (41). In addition, the dissipation rate of indoxacarb was comparatively faster in seasons I and II for tomatoes and cabbages, respectively. This phenomenon may be attributed to the higher rainfall observed in the crop growing period of the respective seasons. For the soil of each season and the associated crops, the dissipation of indoxacarb followed almost the same pattern, with short half-life values ranging between 1.99 and 3.55 d. At harvest, indoxacarb residues were not detected in the soil samples, irrespective of the crop, dose, and season. The overall quick dissipation of the insecticide may be subject to various biotic (microorganisms) and abiotic factors (temperature, humidity, etc.) in the environment (42). The PHI of indoxacarb was found to be in the range of 1.37–4.76 d, irrespective of the dose and season. As the MRL value is mainly a concern for international trade, tomato fruits should be harvested after the mentioned interval. In the case of cabbage, the initial deposit was far enough below the prescribed MRL value for all samples in seasons I and II, so the PHI was not determined. These results are supported by the findings of another research group (22).

### Safety Assessment

The risk quotient values were calculated and are presented in Table 3.

**Table 3:** Dietary and soil ecological risk assessment of indoxacarb in tomato, cabbage, and soil.

**Seasons**	**Treatment**	**Days**	**Dietary risk assessment**						**Soil ecological risk assessment (RQ**_**s**_ **)**			
			**Tomato**			**Cabbage**			**Earthworm**		**Algae**	
			**EDI**	**RQ_**d**_ (Chronic)**	**RQ_**d**_ (acute)**	**EDI**	**RQ_**d**_ (Chronic)**	**RQ_**d**_ (acute)**	**Tomato field soil**	**Cabbage field soil**	**Tomato field soil**	**Cabbage field soil**
Season I	T_1_	0	0.28	0.84	0.04	0.45	1.35	0.07	1.34	2.08	10620.25	16455.70
		1	0.15	0.47	0.02	0.37	1.12	0.05	0.75	1.15	5940.93	9113.92
		3	0.06	0.18	0.01	0.21	0.62	0.03	0.40	0.60	3160.34	4767.93
		7	0.01	0.04	0.00	0.07	0.20	0.01	0.21	0.22	1637.13	1772.15
		10	0.00	0.01	0.00	0.02	0.05	0.00	0.02	0.02	189.87	189.87
		14	-	-	-	0.00	0.01	0.00	-	-	-	-
	T_2_	0	0.48	1.46	0.07	0.79	2.38	0.11	2.44	3.72	19324.89	29409.28
		1	0.24	0.73	0.03	0.62	1.87	0.09	1.48	1.82	11704.64	14430.38
		3	0.09	0.27	0.01	0.32	0.97	0.05	0.93	1.02	7375.53	8101.27
		7	0.02	0.07	0.00	0.12	0.36	0.02	0.37	0.44	2911.39	3502.11
		10	0.00	0.00	0.00	0.03	0.10	0.00	0.16	0.15	1299.58	1181.43
		14	-	-	-	0.00	0.01	0.00	0.07	0.02	578.06	189.87
Season II	T_1_	0	0.28	0.85	0.04	0.47	1.44	0.07	1.37	2.16	10835.44	17071.73
		1	0.23	0.68	0.03	0.34	1.04	0.05	0.69	1.07	5468.35	8430.38
		3	0.11	0.34	0.02	0.18	0.55	0.03	0.51	0.51	4067.51	4071.73
		7	0.05	0.15	0.01	0.05	0.16	0.01	0.32	0.13	2506.33	1033.76
		10	0.00	0.01	0.00	0.01	0.03	0.00	0.14	0.06	1088.61	472.57
		14	-	-	-	0.00	0.01	0.00	0.02	0.02	189.87	189.87
	T_2_	0	0.50	1.50	0.07	0.77	2.34	0.11	2.57	3.89	20358.65	30763.71
		1	0.34	1.02	0.05	0.60	1.80	0.09	1.65	1.69	13029.54	13341.77
		3	0.20	0.60	0.03	0.32	0.98	0.05	1.11	0.89	8789.03	7037.97
		7	0.09	0.28	0.01	0.09	0.27	0.01	0.70	0.27	5561.18	2151.90
		10	0.05	0.15	0.01	0.06	0.20	0.01	0.28	0.12	2185.65	928.27
		14	0.01	0.03	0.00	0.01	0.03	0.00	0.15	0.02	1198.31	189.87

*Indoxacarb: acute 14 d LC_50_ value earthworm - 625 mg kg^−1^; acute 72 h EC_50_ for algae - 0.079 mg l^−1^; PNEC of indoxacarb: earthworm – 0.625 mg kg^−1^, algae – 0.000079 mg l^−1^ (43)*.

#### Dietary Risk Assessment

In the case of tomato crops, the RQ_d_ value for assessing chronic toxicity was below 1 on day 0 of indoxacarb application (at the recommended dose) in both seasons. For double the recommended dose, the corresponding value fell below 1 on days 1 and 3 for seasons I and II, respectively. In cabbage, the same risk quotient value was below 1 on day 3 for both doses and seasons. As far as acute toxicity is concerned, the RQ_d_ value was well below 1 from day 0 onwards, irrespective of the vegetable, dose, and season. Overall, both vegetables may be safely consumed 1 day after indoxacarb application at the recommended dose.

#### Risk Assessment Pertaining to Soil Flora and Fauna

The RQ_s_ values for earthworms were found to be in the low-risk range on the last day of sampling for both vegetables, except for tomatoes in season II at double the recommended dose. However, for algae, all of the values were in the high-risk range. There may therefore be an unacceptably high risk for algae if indoxacarb is applied at the recommended dose; this finding needs to be investigated further.

## Conclusions

A very simple and robust method has been developed to analyse indoxacarb residues in plant and soil samples with acceptable accuracy and precision. The study was important because the experimental location is situated in the leading vegetable-producing state of the country. The results indicate that indoxacarb dissipated quickly in tomatoes, cabbages, and soil, leaving very small amounts of residue in each substrate. The harvested tomatoes and cabbages may safely be consumed 1 day after the final application of the insecticide at the recommended dose. However, to meet the conditions of GAP, a PHI of 3 days should be maintained for tomatoes treated with the insecticide at the recommended dose. Furthermore, the effect of indoxacarb on algae needs to be determined because a high risk is predicted by this study.

## Data Availability Statement

The original contributions presented in the study are included in the article/supplementary material, further inquiries can be directed to the corresponding author.

## Author Contributions

SP, AD, RR, SC, SR, TM, AS, and PG: conceptualization, methodology, validation, investigation, writing—original draft preparation, visualization, and supervision. SP, AD, RR, and PG: software and formal analysis. AD, SC, and AH: resources. SP, PG, AD, RR, and AH: data curation. AMA, AG, MS, MB, and AH: writing—reviewing and editing. AMA, AG, and AH: project administration and funding acquisition. All authors have read and agreed to the published version of the manuscript.

## Funding

This research was funded by Bidhan Chandra Krishi Viswavidyalaya (BCKV), Mohanpur, West Bengal, India. The research programme was also partially supported by the Princess Nourah bint Abdulrahman University Researchers Supporting Project number (PNURSP2022R65), Princess Nourah bint Abdulrahman University, Riyadh, Saudi Arabia.

## Conflict of Interest

The authors declare that the research was conducted in the absence of any commercial or financial relationships that could be construed as a potential conflict of interest.

## Publisher's Note

All claims expressed in this article are solely those of the authors and do not necessarily represent those of their affiliated organizations, or those of the publisher, the editors and the reviewers. Any product that may be evaluated in this article, or claim that may be made by its manufacturer, is not guaranteed or endorsed by the publisher.

## Abbreviations

USA: United States of America
USEPA: United States Environmental Protection Agency
SC: Suspension concentrate
RPM: Revolutions per minute
NCAL: Sodium chloride
Na_2_SO_4_: Sodium sulfate
GC: Gas chromatography
ECD: Electron capture detector
SANTE: RE, Recovery efficiency
RSD: Relative standard deviation
PRSD: Predicted relative standard deviation
Hor: Horwitz ratio
LOD: Limit of detection
LOQ: Limit of quantification
ME: Matrix effect
PE: Process efficiency
PHI: Preharvest interval
MRL: Maximum residue limit
EDI: Estimated daily intake
RQd: Risk quotient
ADI: Acceptable daily intake
EU: European Union
EC: Effect concentration
PNEC: Predicted no effect concentration
GAP: Good agricultural practices.
